# Molecular dynamics simulations based siRNA design against GPR10 reveals stable RNAi therapeutics for hormone-dependent uterine fibroids

**DOI:** 10.1038/s41598-025-16936-z

**Published:** 2025-08-28

**Authors:** Srineevas Sriram, Chandresh Palanichamy, P. T. Subash, Manshi Kumari Gupta, C. Sudandiradoss

**Affiliations:** Department of Biotechnology, School of Biosciences and Technology, Vellore Institute of Technology, Vellore, 632014 Tamil Nadu India

**Keywords:** GPR10, SiRNA, Uterine fibroids, Molecular dynamics, CHARMM-GUI, CHARMM36 force field, RNA interference, Argonaute 2, RNAi therapeutics, In silico drug design, Computational biology and bioinformatics, Reproductive disorders

## Abstract

Uterine fibroids, though benign in nature, are burdensome tumors of the myometrium and continue to weigh heavily on the landscape of women’s health. They affect millions and yet receive a fraction of the therapeutic innovation afforded to malignant diseases. Despite their prevalence, the molecular underpinnings of fibroid pathogenesis have long been met with a blind eye in drug development. Recent insights, however, reveal G-protein-coupled receptor 10 (GPR10) as a central driver of fibroid growth, promoting cell survival through the PI3K/Akt and MAPK/ERK signaling pathways following REST repression. In this study, we present a rigorous, computationally guided approach to design small interfering RNAs (siRNAs) that silence GPR10 expression at the transcriptomic level. Beginning with a library of 275 siRNA candidates, we undertook a layered in-silico refinement process, combining thermodynamic assessment, secondary structure modeling and off-target filtration, to distill a shortlist of ten high-confidence molecules. These were subjected to structural docking against Argonaute 2, the catalytic engine of the RNA-induced silencing complex, revealing siRNA8 and siRNA12 as lead candidates distinguished by robust binding affinity, high predicted silencing efficacy, which was greater than 93.5%, and precise conformational fit. Subsequent molecular dynamics simulations under CHARMM-GUI/CHARMM36m force field, confirmed the structural stability and sustained silencing potential of the complex. Collectively, these findings identify GPR10 as a therapeutically actionable driver in fibroid biology and lay the groundwork for precision RNAi strategies targeting non-malignant, yet clinically neglected, hormone-dependent disorders.

**Supplementary Information:**

The online version contains supplementary material available at 10.1038/s41598-025-16936-z.

## Introduction

 Uterine fibroids (UF), also known as uterine leiomyomas, have been extensively documented in medical literature for centuries, historically described as uterine stones before their formal classification in the 19th century^1^. These benign smooth muscle tumors originate within the myometrium and are the most prevalent pelvic neoplasms among reproductive-age women, with an estimated incidence of up to 75%^2^. Despite their benign nature, UF are a major source of gynecological morbidity, often leading to symptoms such as abnormal uterine bleeding, pelvic pressure, pain, and infertility. Their impact extends beyond individual health, posing a significant economic burden due to healthcare costs and lost productivity. Histologically, fibroids are characterized by excessive extracellular matrix deposition and disorganized smooth muscle cell proliferation, which contribute to their pathogenesis^3^. Although hormonal factors, particularly estrogen and progesterone, play a critical role in UF growth, emerging evidence suggests that dysregulated signaling pathways and aberrant gene expression patterns also contribute to fibroid development and progression.

Recent studies have implicated G-protein-coupled receptor 10 (GPR10), also known as the prolactin-releasing peptide receptor (PRLHR), in the pathogenesis of UF. Originally characterized for its role in prolactin regulation within the hypothalamus, GPR10 has since been identified as highly overexpressed in fibroid tissues, suggesting a functional role in tumor development^4^. GPR10 activation by prolactin-releasing peptide (PrRP) triggers multiple intracellular signaling cascades, including the phosphatidylinositol 3-kinase (PI3K)/Akt and mitogen-activated protein kinase (MAPK)/extracellular signal-regulated kinase (ERK1/2) pathways, both of which are known to promote cell proliferation and inhibit apoptosis^5^. These pathways contribute to increased fibroid cell survival, enhanced extracellular matrix production, and resistance to programmed cell death, which are hallmarks of UF pathology. Furthermore, dysregulation of GPR10 has been linked to the loss of transcriptional repression mechanisms that would normally control its expression, allowing for its unchecked activity in fibroid tissues^4^.

GPR10 primarily signals through the Gαq/11 pathway, which leads to the activation of phospholipase C (PLC), hydrolysis of phosphatidylinositol 4,5-bisphosphate (PIP2), and the subsequent production of inositol triphosphate (IP3) and diacylglycerol (DAG)^6^. This cascade results in intracellular calcium mobilization, a process implicated in smooth muscle contractility and cell signaling.

Additionally, GPR10 has been shown to activate the MAPK/ERK1/2 pathway, which is associated with increased cell proliferation and survival. Persistent activation of ERK1/2 in UF has been linked to excessive fibroblast-like cell behavior and heightened ECM production, leading to tumor progression^5^. A study published by Maletínská et al. (2011)^7^ investigated prolactin-releasing peptide (PrRP) analogs and found that certain analogs, such as [PheNO_2_^31^]PrRP20 and [Tyr^31^PrRP20, exhibited high affinities for the GPR10 receptor. These analogs effectively activated MAPK/ERK1/2 and CREB signaling pathways, resulting in significant anorexigenic effects in fasted mice. A 2010 study by Maixnerová et al. (2010)^8^ characterized the binding and signaling properties of PrRP and reported that PrRP binding to GPR10 leads to the activation of multiple signaling pathways, including MAPK/ERK1/2 and cAMP response element-binding protein (CREB).

**Fig. 1:**
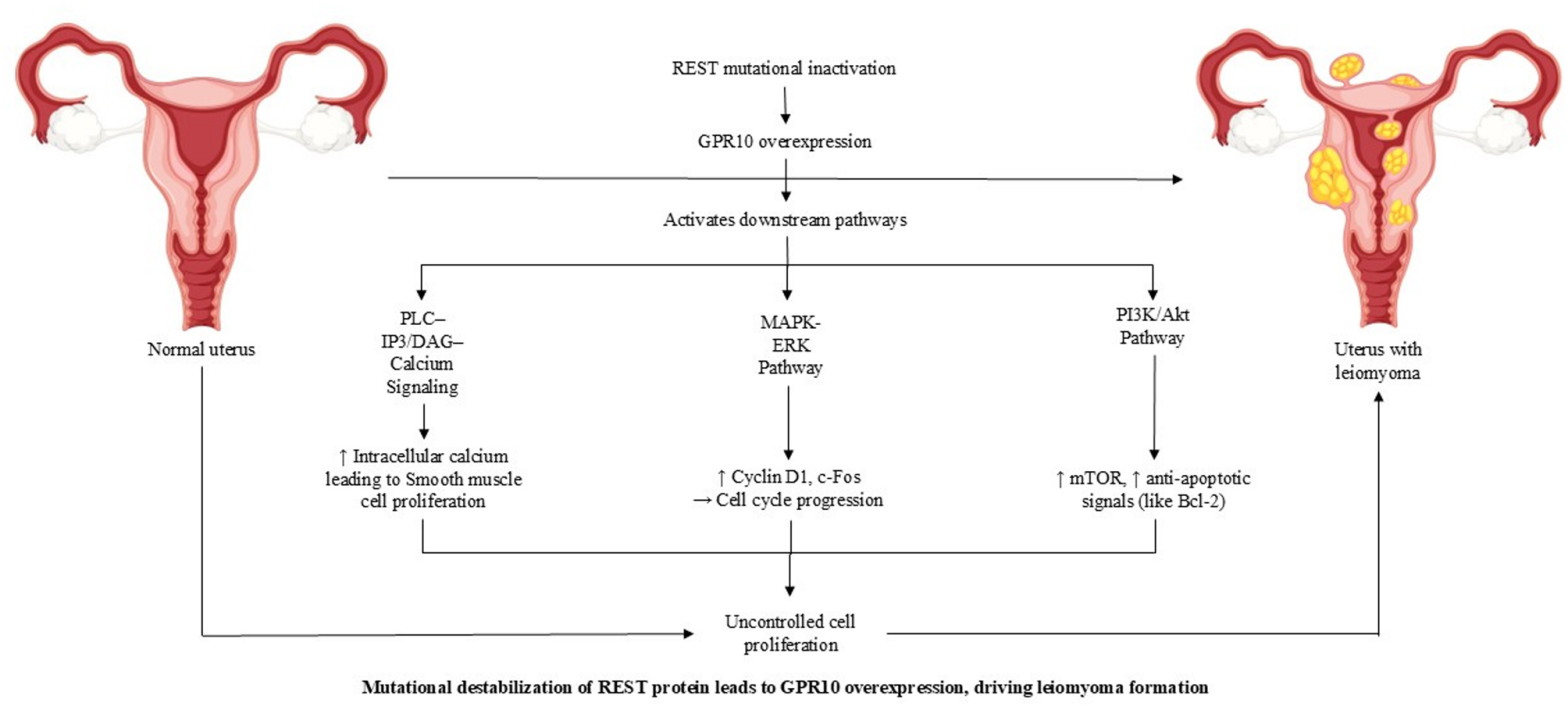
GPR10 overexpression activates multiple oncogenic signaling pathways contributing to uterine leiomyoma formation. GPR10 is markedly upregulated in leiomyomatous tissue and serves as a central mediator of pathological signaling. Its overexpression triggers downstream activation of the PLC-IP3/DAG-calcium signaling cascade, leading to increased intracellular calcium and smooth muscle cell proliferation. Simultaneously, GPR10 stimulates the MAPK-ERK and PI3K-Akt pathways, promoting cell cycle progression via upregulation of Cyclin D1 and c-Fos, and enhancing anti-apoptotic signaling through mTOR and Bcl-2. These synergistic effects result in uncontrolled proliferation of uterine smooth muscle cells, driving fibroid development.

Another important signaling pathway activated by GPR10 is the PI3K/Akt pathway^5^which promotes cell survival by inhibiting apoptosis and supporting metabolic adaptation under stress conditions. Notably, the repressor element 1-silencing transcription factor (REST), a tumor suppressor, has been shown to regulate GPR10 expression under normal conditions. Loss of REST, as observed in uterine fibroids (UF), leads to aberrant overexpression of GPR10, thereby facilitating uncontrolled activation of the PI3K/Akt pathway^4^. Increased Akt activity has been observed in UF, where it enhances resistance to apoptosis, which is a key feature distinguishing fibroid cells from normal myometrial cells. This resistance to programmed cell death is thought to contribute to fibroid persistence and recurrence, making GPR10 a central player in UF pathophysiology^9–12^. These interconnected signaling mechanisms activated by GPR10 are summarized in Fig. 1, which illustrates how its overexpression drives multiple oncogenic pathways contributing to uterine leiomyoma formation.

Given the central role of GPR10 and the PI3K/Akt pathway in fibroid pathophysiology, therapeutic strategies aimed at silencing GPR10 expression, such as RNA interference, have garnered significant attention. RNA interference (RNAi) has emerged as a highly effective tool for selectively silencing gene expression and holds promise as a therapeutic approach for fibroid treatment^13^. Small interfering RNAs (siRNAs), a class of short double-stranded RNA molecules, facilitate this process by guiding the RNA-induced silencing complex (RISC) to degrade complementary messenger RNA (mRNA) transcripts, thereby preventing translation and reducing protein expression^14^.

**Fig. 2:**
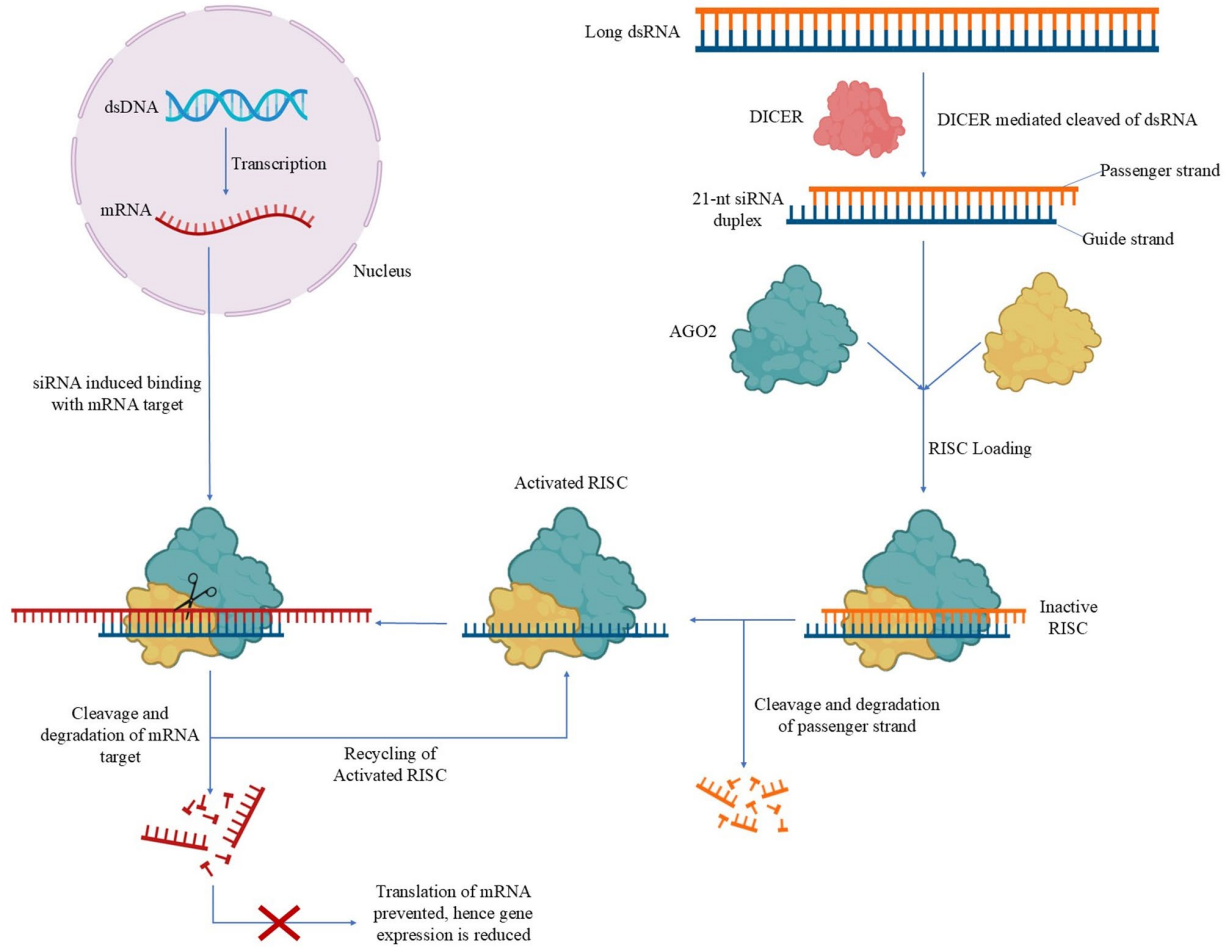
Mechanism of RNA interference (RNAi) via siRNA-loaded RISC complex. Long double-stranded RNA (dsRNA) is processed by the Dicer enzyme into ~ 21-nucleotide siRNA duplexes with 2-nucleotide 3′ overhangs. The duplex is loaded onto Argonaute 2 (Ago2) during RISC assembly, forming an inactive RISC. The passenger strand is then cleaved and degraded, yielding an activated RISC containing Ago2 and the guide strand. This complex binds to complementary target mRNA, leading to mRNA cleavage and degradation. As a result, translation is inhibited and gene expression is reduced. Activated RISC is recycled for repeated silencing activity.

siRNA molecules are typically 21–23 nucleotides in length with 2-nucleotide 3′ overhangs and act through the conserved RNAi pathway in eukaryotic cells. Upon entry into the cytoplasm, siRNA is either processed from longer double-stranded RNA by the RNase III enzyme Dicer or introduced directly as a synthetic duplex^15,16^. The duplex is then loaded into the RNA-induced silencing complex (RISC), where it undergoes strand separation. During this process, the guide strand (antisense) is selectively retained and incorporated into the active RISC, while the passenger strand (sense) is degraded^15,17^. This assembly process is facilitated by the TAR RNA-binding protein (TRBP), which acts as a cofactor of Dicer and plays a key role in recruiting and stabilizing the siRNA duplex for efficient loading onto Argonaute proteins. TRBP also helps bridge interactions between Dicer and AGO2, contributing to the handoff of the guide strand to RISC. Central to RISC function are Argonaute proteins, particularly AGO2, which serves as the primary catalytic engine of gene silencing. While multiple Argonaute family members exist in humans (AGO1-AGO4), only AGO2 possesses endonucleolytic activity necessary for mRNA cleavage. AGO2 contains several conserved domains critical to its function. The PAZ domain binds the 3′ overhang of the siRNA guide strand, anchoring it in position, while the MID domain secures the 5′ phosphate end, orienting the siRNA within the complex^15^. Together, these domains enable accurate positioning of the guide strand, allowing AGO2 to base-pair with the target mRNA and catalyze its cleavage at the center of the complementary region. This precise architecture ensures efficient and specific degradation of the target transcript, halting translation and reducing protein expression^17^. Figure 2 illustrates this siRNA mediated silencing mechanism.

Despite its precision, the therapeutic application of siRNA faces several delivery-related challenges. siRNAs are inherently unstable in serum and susceptible to degradation by nucleases. Additionally, their negative charge and hydrophilicity limit cellular uptake. To address these limitations, siRNAs are frequently chemically modified or encapsulated in delivery vehicles such as lipid nanoparticles or polymer-based carriers^17^. These delivery systems enhance serum stability, facilitate cellular uptake, promote endosomal escape, and ultimately ensure efficient cytoplasmic release, thereby maximizing gene silencing efficacy while minimizing off-target effects.

Unlike small-molecule inhibitors or monoclonal antibodies, siRNAs offer a more specific approach to gene silencing, with potential to minimize off-target effects and systemic toxicity^18^when carefully designed. The use of siRNA-based therapeutics has gained substantial interest for its potential to selectively target aberrantly expressed genes such as GPR10 in UF^19^. However, designing an effective siRNA requires careful optimization, as factors such as GC content, thermodynamic stability, and mRNA secondary structure influence silencing efficiency. In addition, off-target interactions and immune activation remain important design consideration and must be addressed to fully harness the therapeutic precision of siRNAs and ensure its safety^20^.

Recent advancements in computational tools such as molecular docking, molecular dynamics simulations, and structure-based modeling have significantly improved the design and prediction of siRNA efficacy. These methods enable the identification of siRNA sequences with high target specificity and reduced risk of off-target binding, thereby enhancing both potency and therapeutic reliability^21^.

In this study, we aimed to design and evaluate siRNAs targeting GPR10 to achieve efficient and specific gene knockdown in uterine fibroids. Using computational prediction tools, we screened potential siRNA candidates based on key sequence parameters, including GC content, binding energy, and off-target potential. Further refinement involved molecular docking and molecular dynamics simulations to assess siRNA interactions with the RISC complex, ensuring efficient target recognition and degradation. By integrating computational modeling with predictive validation techniques, we sought to identify highly specific and potent siRNAs capable of downregulating GPR10. This work contributes to the broader effort of developing RNAi-based strategies for targeted gene regulation and provides insights into novel therapeutic approaches for Uterine Fibroids.

## Results and discussion

### Dataset collection

NCBI’s Nucleotide database^22^ was used to retrieve the FASTA format of the coding DNA sequence (CDS) of the human GPR10 mRNA. The retrieved transcript, with the accession number NM_004248.3, is a linear mRNA consisting of 5387 base pairs. This sequence served as the foundational input for the in-silico design of small interfering RNA (siRNA) molecules aimed at silencing GPR10 gene expression.

To simulate siRNA loading and assess potential interactions within the RNA-induced silencing complex (RISC), the structure of Argonaute2 (AGO2) was retrieved. AGO2 is the central catalytic component of the RISC complex. The sequence and structural information for AGO2 was obtained from the UniProt database (Accession: Q9UKV8), which consists of 859 amino acids. The AlphaFold-predicted 3D structure^23,24^ of AGO2 complements this validated sequence information and was used in downstream computational studies to model the binding of siRNA candidates to the PAZ and MID domains, which are responsible for siRNA recognition, anchoring, and cleavage facilitation. To validate the reliability of the structural model, residue-wise confidence scores (pLDDT) were analyzed and visualized as shown in Fig. 5c. The functionally critical PAZ and MID domains exhibit very high confidence (pLDDT > 90), indicating a high level of structural accuracy in these regions. Regions with lower confidence scores (pLDDT < 50) were primarily limited to some N-terminal and flexible loop segments. Overall, the predicted structure had an average pLDDT score of 92.39.

Although high-resolution crystallographic structures of AGO2, such as PDB ID: 4OLA^25,26^are available, they contain multiple unresolved regions, including residues 1–22 at the N-terminus, several internal loop segments, and residues 818–838 at the C-terminal end (as noted in the REMARK 465 of its PDB file).

### Designing candidate siRNAs for GPR10 silencing

A total of 275 siRNA candidates were generated against the human GPR10 mRNA sequence using the siDirect 2.0 webserver^27^. Among these, 23 siRNAs were shortlisted based on their compliance with all three functional siRNA design algorithms, Ui-Tei^28^Reynolds^29^ and Amarzguioui^30^. Additional stringent filtering parameters such as a GC content range of 30–55% and a maximum seed duplex melting temperature of 21.5 °C were applied to ensure high silencing efficiency while minimizing off-target effects. This Tm threshold follows the design criteria established by Ui-Tei et al. (2008)^31^which showed that low thermodynamic stability in the seed region reduces the likelihood of miRNA-like off-target interactions at physiological temperature (~ 37 °C).

The selected 23 siRNAs demonstrated optimized characteristics across multiple design criteria. The GC content ensured a balanced thermodynamic profile, enhancing the probability of efficient incorporation into the RNA-induced silencing complex (RISC) while also reducing the risk of non-specific binding to off-target transcripts. It is worth noting that a high GC content, although beneficial for stable target binding, may increase non-specific hybridization; the applied GC range successfully circumvented this issue. Each of the selected siRNAs displayed a minimum number of partial mismatches of 1 when aligned with the rest of the transcriptome, and no siRNA showed more than one perfect match, thus significantly minimizing the risk of off-target silencing. This indicates high specificity in target recognition, which is critical for effective gene silencing in experimental and therapeutic settings.

Supplementary Table 1 summarizes the target position, guide strand, target sequence, and passenger strand of each of the final 23 functional siRNAs. These siRNAs cover diverse regions of the GPR10 transcript, from early to late coding regions, thereby increasing the likelihood of successful transcript knockdown regardless of potential secondary structures in mRNA that might hinder siRNA accessibility. The comprehensive approach employed in this study demonstrates the utility of combining multiple siRNA design principles with thermodynamic and off-target filtering criteria. The identified siRNAs are ideal candidates for future in vitro or in vivo silencing studies of GPR10, particularly in therapeutic and functional genomics research. Future work includes validating the efficacy of these siRNAs to confirm their knockdown efficiency and specificity.

### Structural and thermodynamic analysis of siRNA

A comprehensive computational evaluation was carried out to identify optimal siRNA candidates targeting the GPR10 mRNA, focusing on ten shortlisted siRNA candidates, siRNA3, siRNA6, siRNA8, siRNA10, siRNA12, siRNA13, siRNA14, siRNA17, siRNA18, and siRNA23. These candidates were assessed based on structural characteristics, thermodynamic properties, hybridization potential, melting behavior and predicted silencing efficacy.

**Fig. 3:**
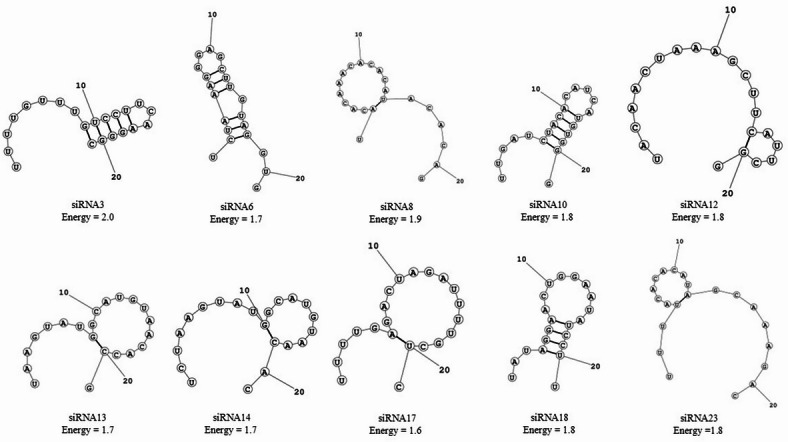
Predicted secondary structures of selected siRNA candidates using MaxExpect. Secondary structures of siRNA3, siRNA6, siRNA8, siRNA10, siRNA12, siRNA13, siRNA14, siRNA17, siRNA18, and siRNA23 were generated based on minimum free energy predictions. Each structure is annotated with its corresponding free energy value (in kcal/mol), highlighting the conformational diversity and thermodynamic stability among siRNA designs. Lower energy values correspond to more thermodynamically stable conformations.

The secondary structures of the guide strands were first predicted using the MaxExpect tool from the RNAstructure web server^32^ as shown in Fig. 3. All selected siRNAs exhibited relatively low structural stability, with minimal internal base pairing and free energy values ranging from 1.6 to 2.0 kcal/mol. This low propensity for intramolecular folding is advantageous, as excessive internal pairing could hinder strand loading into the RNA-induced silencing complex (RISC). The presence of flexible, largely unstructured siRNA guide strands, particularly in siRNA3, siRNA8 and siRNA12 indicated that these candidates are well-suited for efficient incorporation into the silencing complex.

Further validation using the Mfold^33^ tool corroborated the MaxExpect predictions, showing no significantly stable secondary structures that might impede the function of the guide strands. The siRNAs retained predominantly linear configurations with small, energetically permissible loop or stem regions, maintaining thermodynamic profiles conducive to effective gene silencing.

**Fig. 4:**
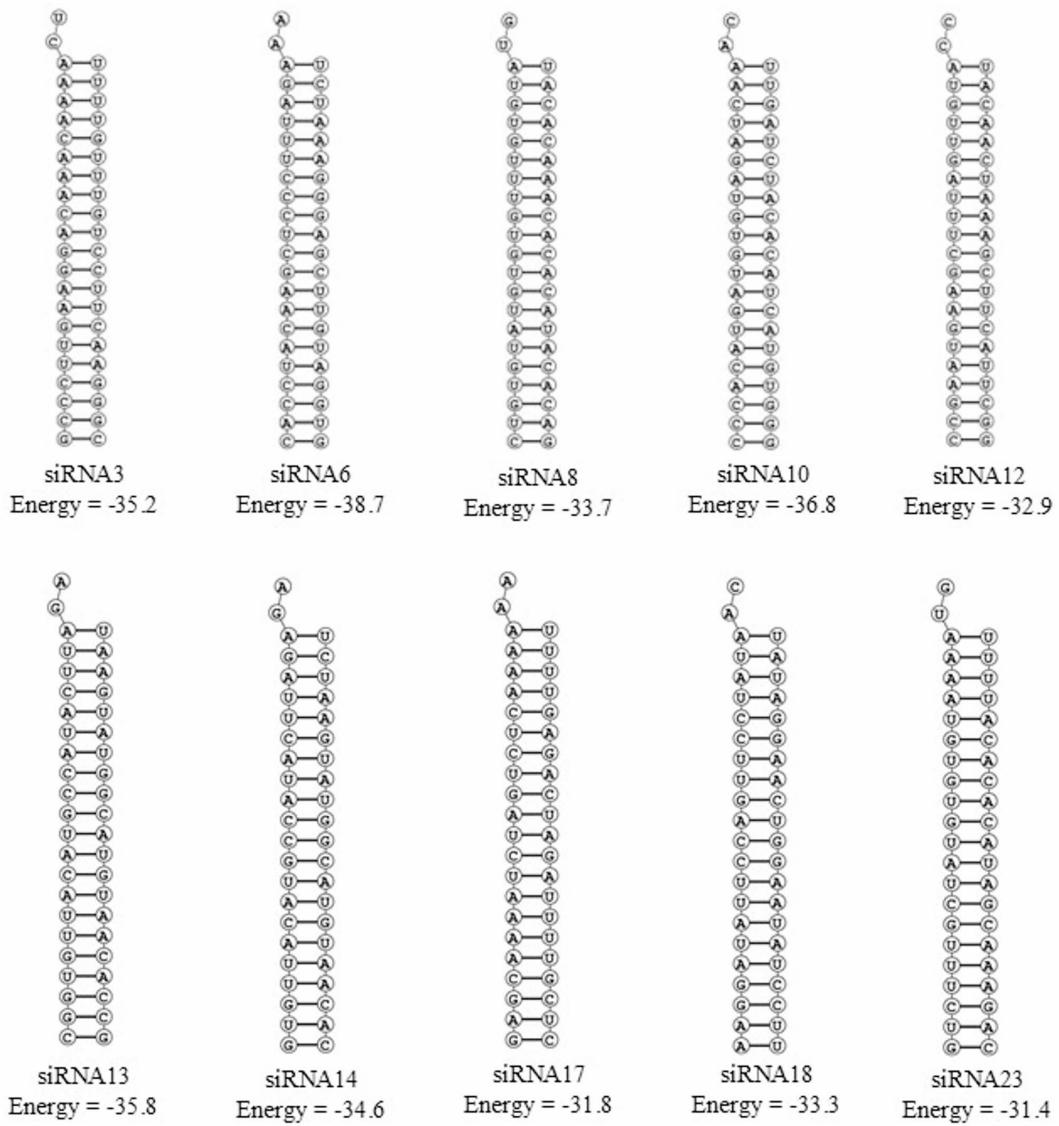
Predicted secondary structures and free energies of siRNA duplexes targeting GPR10 mRNA. Ten siRNA candidates were evaluated for thermodynamic stability using RNAstructure’s DuplexFold algorithm. The secondary structures shown correspond to siRNA3, siRNA6, siRNA8, siRNA10, siRNA12, siRNA13, siRNA14, siRNA17, siRNA18, and siRNA23. Calculated minimum free energy (ΔG) values range from − 38.7 kcal/mol (siRNA6) to −31.4 kcal/mol (siRNA23), with more negative values indicating greater duplex stability.

The DuplexFold^32^ module was employed to analyze the hybridization between each siRNA guide strand and its complementary target region within the GPR10 mRNA. Figure 4 shows that all candidate duplexes demonstrated favorable binding thermodynamics, with negative ΔG values indicating spontaneous and stable hybrid formation. Among these, siRNA6 (−38.7 kcal/mol), siRNA10 (−36.8 kcal/mol) and siRNA13 (−35.8 kcal/mol) formed the most stable RNA-RNA duplexes, suggesting strong mRNA target binding strength and hence a high silencing potential.

To assess the temperature-dependent stability of these duplexes, melting profiles were generated using the DINAMelt^34^ tool. The predicted melting temperatures (Tm (conc)) for all siRNA-mRNA hybrids ranged from 82.9 °C to 90.9 °C, all well above the physiological temperature, indicating strong duplex stability that would persist in vivo. While siRNA-mRNA duplexes do not operate at elevated temperatures in vivo, high Tm values reflect robust hybridization affinity that ensures the duplex remains stable at 37 °C, supporting effective target binding and silencing. Among the candidates, siRNA6 showed the highest guide strand Tm (conc) at 90.9 °C, followed by siRNA3 (88.6 °C) and siRNA10 (86.7 °C), supporting their superior thermodynamic robustness. High Tm values are indicative of the potential for prolonged mRNA engagement and sustained silencing effects.

An analysis of GC content^35^ revealed that all the selected siRNAs maintained GC percentages within the optimal range (typically between 30% and 55%), balancing the need for duplex stability without compromising target specificity. siRNA6 (48%), siRNA3 (43%), siRNA10 (43%) and siRNA13 (43%) in particular exhibited highly favorable GC compositions, contributing to their effective hybridization characteristics. The folding free energy (ΔG) values for the single-stranded guide strands, computed using the Oligonucleotide Properties Calculator^35^ranged from − 22.5 to −26.1 kcal/mol. These values suggest that while the siRNAs possess a degree of structural stability to resist nuclease degradation, they are not overly thermostable, allowing for efficient RISC loading.

Functional efficacy was predicted using the OligoWalk tool^36^which estimates the likelihood of successful gene silencing based on a combination of binding energy, target accessibility, and duplex formation thermodynamics. The results revealed siRNA12 as the top-performing candidate, with the highest predicted efficacy percentage of 96.1845%. Close behind were siRNA10 and siRNA17 all surpassing the 92% threshold, indicating a high probability of effective gene silencing in biological systems. Although siRNA6 and siRNA14 exhibited slightly lower efficacy predictions, they still fell within a functionally relevant range and could be considered viable in alternative or combination-based strategies.

In summary, the integration of multiple in silico parameters enabled the identification of a subset of 5 highly promising siRNAs, with siRNA3, siRNA6, siRNA8, siRNA10 and siRNA12 emerging as the most effective candidates; their results are summarized in Table 1. These guide strands demonstrated favorable structural flexibility, strong target binding potential, robust thermodynamic profiles, and high predicted silencing efficacy. The remaining candidates, such as siRNA14 and siRNA17, also met several key criteria and may serve as valuable alternatives or complements in downstream functional assays. This rational, data-driven selection provides a strong foundation for further experimental validation and potential therapeutic application targeting GPR10.

**Table 1:** Biophysical properties and structural-thermodynamic parameters of the top 5 shortlisted SiRNA candidates via sidirect targeting GPR10 mRNA. **Abbreviations**: Tm — melting temperature; Cp — constant pressure; Conc — concentration; ΔG — Gibbs free energy; GC — guanine-cytosine content.

siRNA name	mRNA target position	mRNA target sequence (5’→3’)	siRNA guide strand (5’→3’)	siRNA passenger strand (5’→3’)	Seed-Duplex Stability		GC % content	Tm (Conc)	Tm(Cp)	Thermodynamic Constant ΔG (kcal/mol)	Free Energy of Binding (kcal/mol)	Free Energy of Folding (kcal/mol)	siRNA Efficacy (%)
					Guide Tm (°C)	Passenger Tm(°C)							
siRNA3	1514–1536	GCCCTTGAAGGACAAACAAAACT	UUUUGUUUGUCCUUCAAGGGC	CCUUGAAGGACAAACAAAACU	5.6	16.6	43%	88.6 °C	89.8 °C	−26.1	−35.2	2	93.2486
siRNA6	1861–1883	CACCTACAAGCTCCCTTTAGAAA	UCUAAAGGGAGCUUGUAGGUG	CCUACAAGCUCCCUUUAGAAA	15.8	16.4	48%	90.9 °C	92.1 °C	−25.9	−38.7	1.7	91.0699
siRNA8	2031–2053	CTGTGTATGTGTGTTTGTGTATG	UACACAAACACACAUACACAG	GUGUAUGUGUGUUUGUGUAUG	19.3	20.4	38%	84.9 °C	86.1 °C	−24	−33.7	1.9	93.4435
siRNA10	2308–2330	CCCACATGATGTGTAGATCAAAC	UUGAUCUACACAUCAUGUGGG	CACAUGAUGUGUAGAUCAAAC	21.4	20.5	43%	86.7 °C	87.8 °C	−24.7	−36.8	1.8	95.2393
siRNA12	3722–3744	CCGAATGAAGCTTTAGTTGTACC	UACAACUAAAGCUUCAUUCGG	GAAUGAAGCUUUAGUUGUACC	19	12	38%	82.9 °C	84.2 °C	−24.8	−32.9	1.8	96.1845

### Docking of siRNA with Argonaute 2 protein

The ten shortlisted siRNA candidates for targeting the GPR10 mRNA: siRNA3, siRNA6, siRNA8, siRNA10, siRNA12, siRNA13, siRNA14, siRNA17, siRNA18, and siRNA23, where further subjected for molecular docking analysis with the AGO2 protein. This was performed using the HDOCK web server^37^ which provided a docking score, where a more negative binding score represents a stronger binding affinity between the siRNA and AGO2 protein. A confidence score showing the likelihood of the 2 molecules to bind is also provided for each complex.

As listed in Table 2, all the ten shortlisted siRNAs exhibited very high affinity with the AGO2 protein, given that a typical RNA-protein complex normally has a docking score of around − 200 and the input complexes in this analysis had a docking score in the range of −282.17 to −370.24. siRNA8, siRNA3 and siRNA6 had the top 3 most negative docking scores of −370.24, −361.14 and − 359.67, respectively while each having confidence greater than 98%. From this it can be inferred that these 3 siRNA candidates will have the most efficient RISC loading as the siRNA can stably bind to the AGO2, particularly to the PAZ and MID domains that anchor the 3’ and 5’ ends of the guide strand.

**Table 2:** Molecular Docking results of the 10 shortlisted SiRNAs targeting GPR10 mRNA.

siRNA name	mRNA target position	mRNA target sequence (5’→3’)	siRNA guide strand (5’→3’)	siRNA passenger strand (5’→3’)	Docking Score	Confidence (%)
siRNA3	1514–1536	GCCCTTGAAGGACAAACAAAACT	UUUUGUUUGUCCUUCAAGGGC	CCUUGAAGGACAAACAAAACU	−361.14	98.56
siRNA6	1861–1883	CACCTACAAGCTCCCTTTAGAAA	UCUAAAGGGAGCUUGUAGGUG	CCUACAAGCUCCCUUUAGAAA	−359..67	98.51
siRNA8	2031–2053	CTGTGTATGTGTGTTTGTGTATG	UACACAAACACACAUACACAG	GUGUAUGUGUGUUUGUGUAUG	−370.24	98.79
siRNA10	2308–2330	CCCACATGATGTGTAGATCAAAC	UUGAUCUACACAUCAUGUGGG	CACAUGAUGUGUAGAUCAAAC	−293.61	94.65
siRNA12	3722–3744	CCGAATGAAGCTTTAGTTGTACC	UACAACUAAAGCUUCAUUCGG	GAAUGAAGCUUUAGUUGUACC	−348.68	98.15
siRNA13	3780–3802	CGGTGTTACATGCCATACTTAGA	UAAGUAUGGCAUGUAACACCG	GUGUUACAUGCCAUACUUAGA	−321.62	96.87
siRNA14	3782–3804	GTGTTACATGCCATACTTAGAGA	UCUAAGUAUGGCAUGUAACAC	GUUACAUGCCAUACUUAGAGA	−324.48	97.04
siRNA17	4245–4267	GAGCAAAATCTAGTCTCAAAAAA	UUUUGAGACUAGAUUUUGCUC	GCAAAAUCUAGUCUCAAAAAA	−331.77	97.43
siRNA18	4973–4995	AAGGATATTCCAGTTCCTATAAC	UAUAGGAACUGGAAUAUCCUU	GGAUAUUCCAGUUCCUAUAAC	−282.17	93.36
siRNA23	5273–5295	GTCTTTGCTATGTGTGTAAAATG	UUUUACACACAUAGCAAAGAC	CUUUGCUAUGUGUGUAAAAUG	−320.26	96.79

**Fig. 5:**
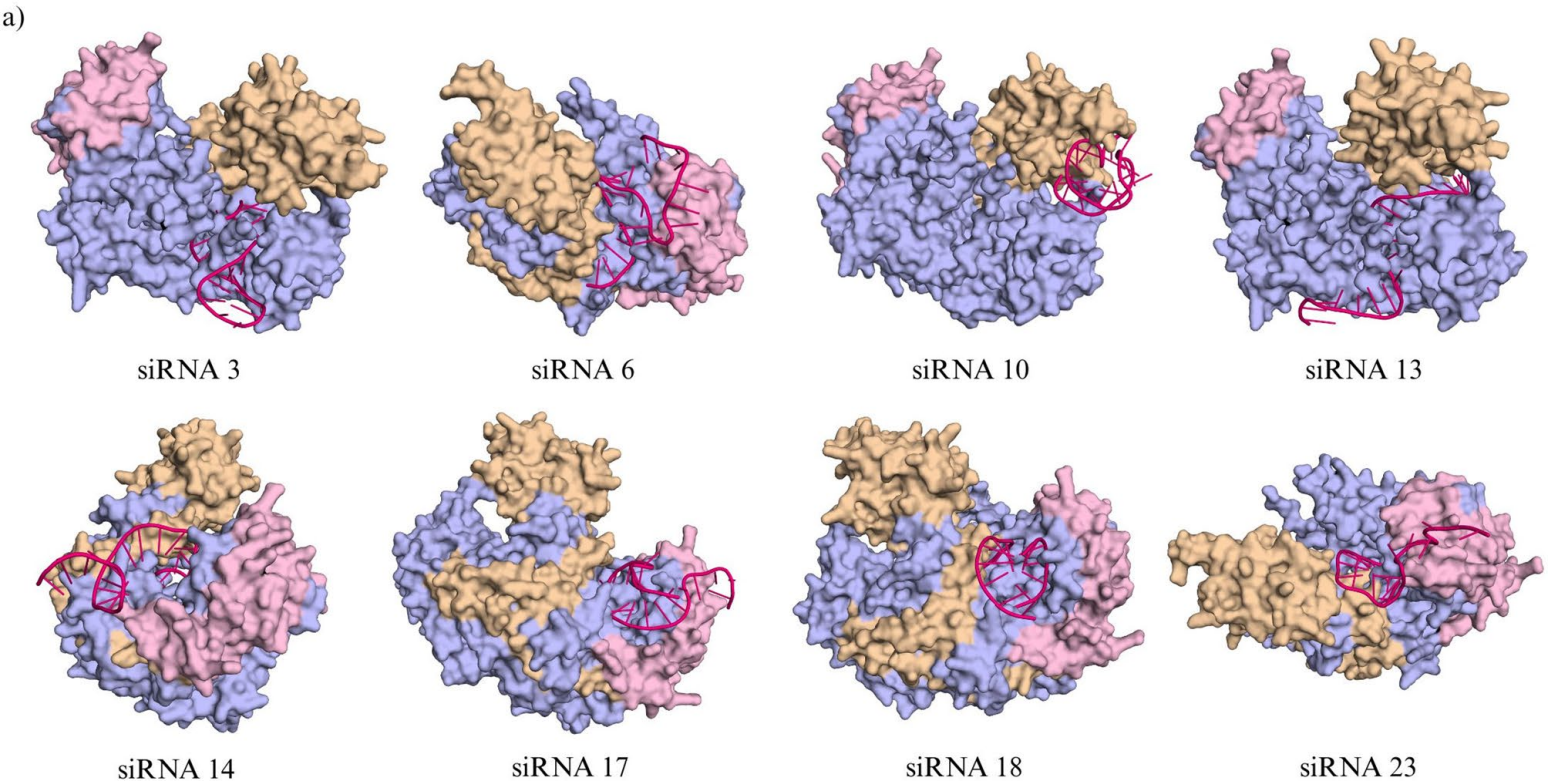

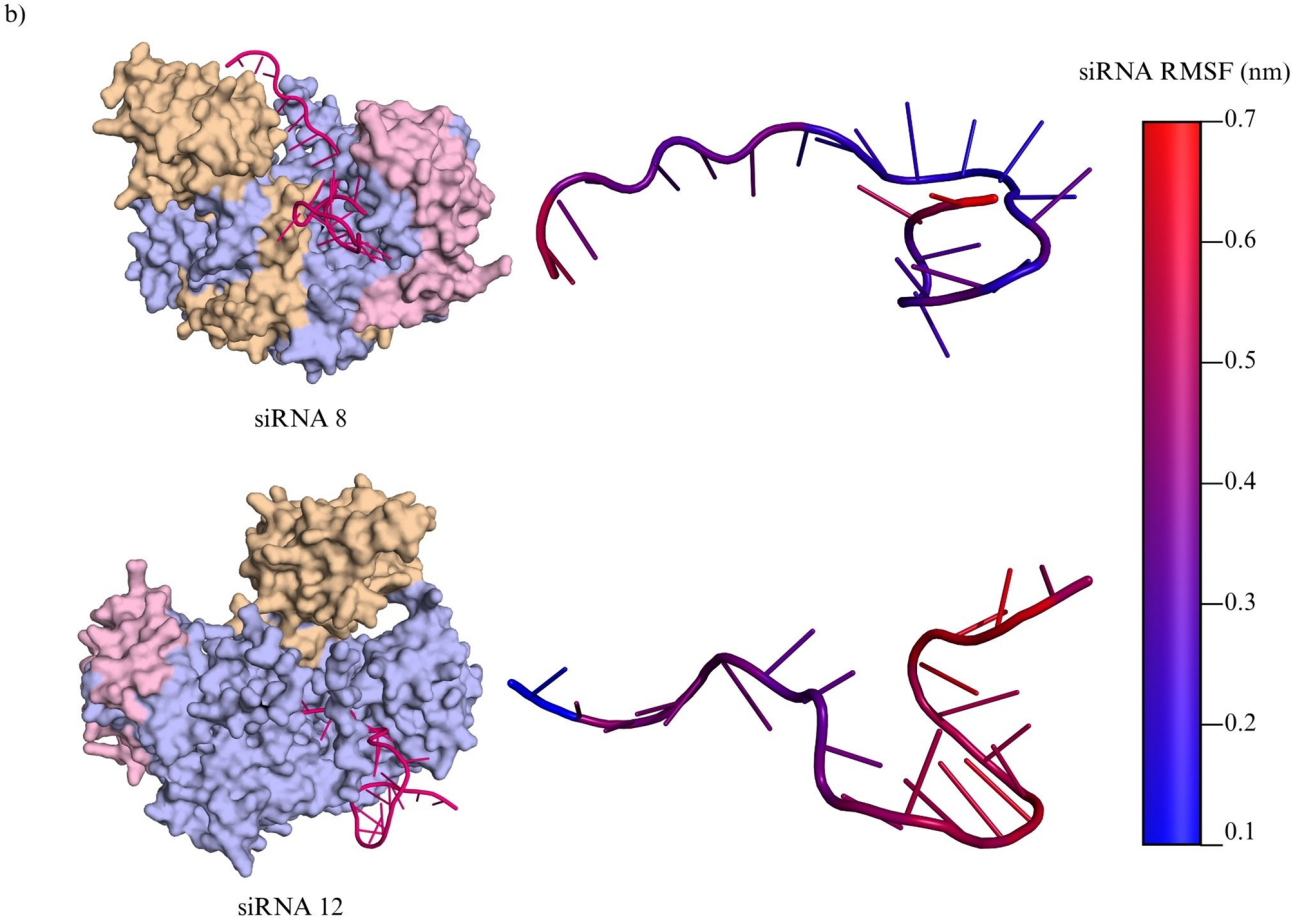

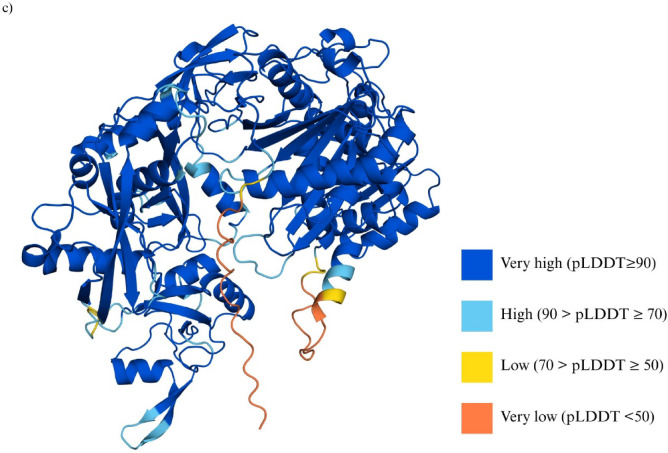
Structural characterization of AGO2-siRNA interactions through molecular docking and MD simulations. (**a**) Molecular docking of selected siRNA candidates with the Argonaute protein (AGO2). Surface representations illustrate the binding conformations of siRNAs (magenta) within the Argonaute complex, with its PAZ domain (light brown) and MID domain (light pink). Each siRNA adopts a unique orientation and interaction pattern, indicating potential variability in Argonaute loading efficiency and RISC complex formation. (**b**) Molecular dynamics (MD) simulation of top-ranked docked complexes for siRNA8 and siRNA12. The left panels show the docked AGO2-siRNA complexes, while the right panels depict the siRNA structures color-mapped according to root mean square fluctuation (RMSF) values (in nm), highlighting nucleotide-level flexibility. Increased RMSF values (red) indicate regions of higher structural mobility, potentially influencing target recognition or silencing efficiency. (**c**) Per-residue confidence scores (pLDDT) of the AlphaFold-predicted AGO2 structure (UniProt ID: Q9UKV8). The ribbon representation is color-coded according to pLDDT scores: very high confidence (≥ 90) in dark blue, high (70–89) in light blue, low (50–69) in yellow, and very low (< 50) in orange.

Following the docking analysis, each of the siRNA-AGO2 docked complexes were visualized using PyMOL^38^as shown in Fig. 5, to assess the orientation of the siRNA within the AGO2 binding pocket. siRNA candidates 3, 10, 13, and 17 were excluded from further analysis due to unfavorable conformational changes observed during docking. These structural shifts led the siRNAs to form intramolecular loops or altered secondary structures, resulting in reduced structural integrity and suboptimal orientation within the AGO2 complex. In particular, siRNA3, 10 and 13 failed to anchor its 3′ and 5′ ends appropriately across the PAZ and MID domains, which is essential for stable RISC loading. siRNA18 was eliminated solely based on its relatively low docking score (− 282.17), despite maintaining structural integrity. This left five siRNAs for final consideration: siRNA6, siRNA8, siRNA12, siRNA14, and siRNA23.

siRNA6 was excluded based on its lowest predicted silencing efficacy (91.07) among the ten candidates. siRNA14 also displayed comparatively lower efficacy (92.27), making both less favorable for downstream evaluation. siRNA23, while structurally acceptable, showed the lowest docking score and confidence score among the remaining pool, further justifying its exclusion.

Ultimately, siRNA8 and siRNA12 retained high docking scores, high predicted efficacy, and ideal structural orientation. Both molecules exhibited correct alignment of their guide strand termini within the PAZ and MID domains of AGO2, maintaining stable and canonical docking conformations essential for efficient RISC loading. Based on this comprehensive in silico evaluation using multiple computational tools, siRNA8 and siRNA12 consistently demonstrated favorable characteristics across all key screening parameters that determine siRNA-mediated gene silencing activity, including thermodynamic stability, strong binding affinity to AGO2 as indicated by their docking scores in HDOCK, and high predicted silencing efficacy. Therefore, these two candidates were selected for molecular dynamics simulations to further assess the structural stability and conformational behavior of their interaction with AGO2.

### Molecular dynamics simulation

The thermodynamic and structural stability of the complex formed by the binding of AGO2 to the chosen siRNA candidates (siRNA8 and siRNA12) was assessed by carrying out a 100 ns molecular dynamics simulation^39^. The key MD trajectory parameters chosen to conduct this comprehensive assessment were Radius of Gyration (Rg), Root Mean Square Deviation (RMSD), Root Mean Square Fluctuation (RMSF), and Potential Energy (PE). The results of these molecular dynamics parameters for the top two siRNA candidates are summarized in Table 3.

**Table 3:** Molecular dynamics results of the best 2 shortlisted SiRNAs (siRNA8, siRNA12) targeting GPR10 mRNA.

siRNA name	mRNA target position	Average RMSD (nm)	Average RMSF (nm)	Average Potential Energy (kJ/mol)	Average Radius of Gyration (nm)
siRNA8	2031–2053	0.460142016	0.221563902	−2191432.758	3.169483187
siRNA12	3722–3744	0.402216039	0.190397079	−2078929.49	3.07525014

**Fig. 6:**
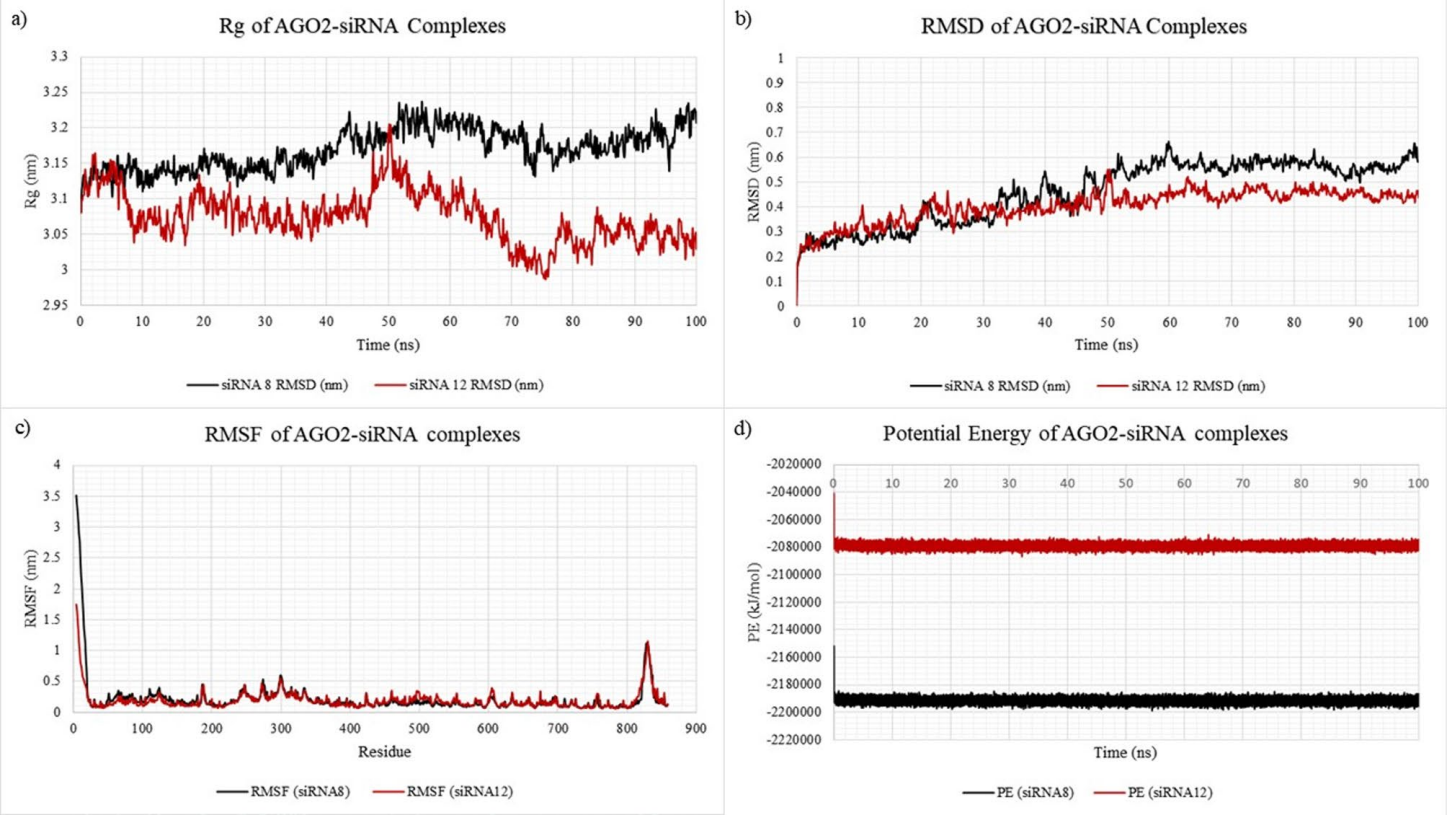
Structural stability and dynamic behavior of AGO2-siRNA complexes over 100 ns molecular dynamics simulations. Comparative analyses of AGO2 which is complexed with siRNA8 (black) and siRNA12 (red) were performed to evaluate conformational stability, flexibility, and interaction energetics. (**a**) Radius of gyration (Rg) plots reveal that siRNA12-bound AGO2 maintains a slightly more expanded conformation compared to siRNA8, which exhibits a gradual decrease in Rg, suggesting higher compactness over time. (**b**) Root mean square deviation (RMSD) profiles indicate that both complexes achieve structural equilibrium, with siRNA8 showing marginally lower deviations, pointing to greater structural stability. (**c**) Root mean square fluctuation (RMSF) analysis shows comparable residue-level flexibility across both complexes, with elevated fluctuations primarily at terminal regions and surface-exposed loops. (**d**) Potential energy (PE) plots demonstrate energetically stable trajectories for both systems, with AGO2-siRNA8 maintaining a lower energy state throughout the simulation, indicative of more favorable binding and complex stabilization.

Figure 6a compares the Rg of both the AGO2-siRNA complexes, which provides insight into the overall compactness during the simulation. The AGO2-siRNA8 complex showed a gradual increase in Rg from approximately 3.13 nm to 3.23 nm by the end of the simulation, indicating a modest conformational relaxation. However, for a protein the size of AGO2 (~ 97 kDa), such a ~ 0.1 nm change falls within the typical fluctuation range observed in molecular dynamics simulations. For example, Paladino et al. (2025)^40^ reported an Rg standard deviation of ~ 0.048 nm for AGO2, and Bhandare and Ramaswamy (2016)^41^ observed AGO2 Rg values ranging between ~ 3.08 and 3.20 nm depending on siRNA interactions. Thus, this shift should be interpreted as a subtle trend in structural behavior, not as a statistically significant expansion. In contrast, the AGO2-siRNA12 complex maintained a relatively stable Rg and even tended to have lower Rg (around 3.05 to 3.10 nm) at points when compared to its early Rg. Together, these Rg profiles suggest that while AGO2-siRNA8 may exhibit slightly greater conformational adaptability, both complexes remain globally stable throughout the simulation.

The RMSD plots as shown in Fig. 6b describe the global structural deviations of the AGO2-siRNA complexes from their original conformation. For AGO2-siRNA8, the RMSD increased steadily from 0.2 nm to a plateau of ~ 0.55–0.6 nm after 60 ns. The average RMSD over the last 40 ns of the simulation was 0.568 nm, indicating a quasi-stable state with moderate conformational rearrangements. Although minor fluctuations remained, the absence of significant structural drift suggests that the system had largely equilibrated by this point. However, AGO2-siRNA12 showed a more stable and lower RMSD (~ 0.4–0.45 nm) throughout the simulation, suggesting less deviation from the starting conformation, indicating that it is more structurally stable than the AGO2-siRNA8 complex. As a result, the more dynamic nature of the AGO2-siRNA8 complex may be biologically relevant, as previous studies have shown that structural flexibility and RNA strain contribute to efficient guide strand binding and RISC loading. Sciabola et al. (2013)^42^ demonstrated that duplex flexibility plays a functional role during antisense strand adsorption into AGO2, a key step in RNA interference. Additionally, Elkayam et al. (2012)^43^ reported that while AGO2 becomes structurally stabilized upon RNA binding, its domains retain the capacity for rearrangement in response to different guide-target interactions, which may facilitate cleavage activity. Together, these findings support the interpretation that moderate structural dynamics, as observed in AGO2-siRNA8, may enhance RISC functionality.

The RMSF plots provide a residue level analysis of flexibility of the AGO2 protein backbone which is complexed with siRNA8 and siRNA12 as shown in Fig. 6c. Both complexes display low fluctuations (less than 0.5 nm) across most residues, indicating that both are generally quite structurally stable. However, a major spike was observed at the N-terminal region (residues 1–20) of both complexes, which corresponds to a low-confidence region in the AlphaFold model (pLDDT < 50) of AGO2, as illustrated in Fig. 5c. These values likely reflect modeling uncertainty rather than meaningful conformational dynamics and therefore were not considered in interpreting global flexibility differences between the two complexes. Around residues 185, 250, 275, 300, and 600, there are also minor spikes that are likely to correspond to surface-exposed loops or domain linkers. A larger spike observed at around residue 825 could indicate heightened mobility at the C-terminal end, possibly corresponding to the PIWI domain or a flexible regulatory loop. However, this region also corresponds to a low-confidence segment in the AlphaFold model (pLDDT < 50), suggesting that the elevated RMSF may be influenced by prediction uncertainty rather than purely intrinsic flexibility. In contrast, the terminal residues (e.g., residues 850–859) exhibit comparatively lower RMSF values, suggesting local stabilization. This behavior is consistent with previous molecular dynamics simulations of human AGO2, where the C-terminal helices (particularly Gln850-Arg854) were shown to reform stable α-helical structures during dynamics, thereby reducing terminal fluctuations (Bhandare & Ramaswamy, 2016)^41^. These findings imply that while the adjacent region may accommodate conformational shifts or suffer from modeling artifacts, the extreme C-terminus remains relatively ordered, potentially aiding in the structural integrity or catalytic alignment of the PIWI domain during siRNA processing. Overall, the RMSF analysis shows that both AGO2-siRNA complexes are structurally stable, with only localized flexibility. AGO2-siRNA8 displays slightly higher fluctuations in loop and linker regions, suggesting modestly greater adaptability. These differences are limited to surface-exposed areas and do not indicate global instability. The combination of local flexibility and a stabilized C-terminus suggests a structural balance in AGO2-siRNA8 that may support efficient siRNA accommodation and silencing.

The thermodynamic stability of the AGO2-siRNA complexes throughout the 100ns simulations were compared and assessed using the PE plots. Figure 6d illustrates the PE plots, which shows that both the complexes reached an equilibrium early in the simulation at ~ 5ns and maintained stable energy profiles without significant fluctuations. The average potential energy of the AGO2-siRNA8 complex was found to be ~ − 2,191,432.76 kJ/mol while the AGO2-siRNA12 complex exhibited a higher average potential energy of ~ − 2,078,929.49 kJ/mol. This relative difference suggests that the AGO2-siRNA8 complex may exhibit a more favorable energetic profile and improved thermodynamic stability under the simulated conditions, although further validation would be needed to assess its statistical significance. The lower potential energy of the AGO2-siRNA8 complex may reflect stronger inter- and intra-molecular interactions, promoting a more stable binding conformation between the AGO2 protein and the siRNA8 guide strand. While potential energy alone does not directly inform on functional persistence, such increased thermodynamic stability could support a more sustained association of the complex. This, in turn, may favor enhanced gene silencing activity by potentially facilitating more consistent target recognition and cleavage over time. However, this hypothesis would require further kinetic or functional validation to be confirmed.

**Fig. 7:**
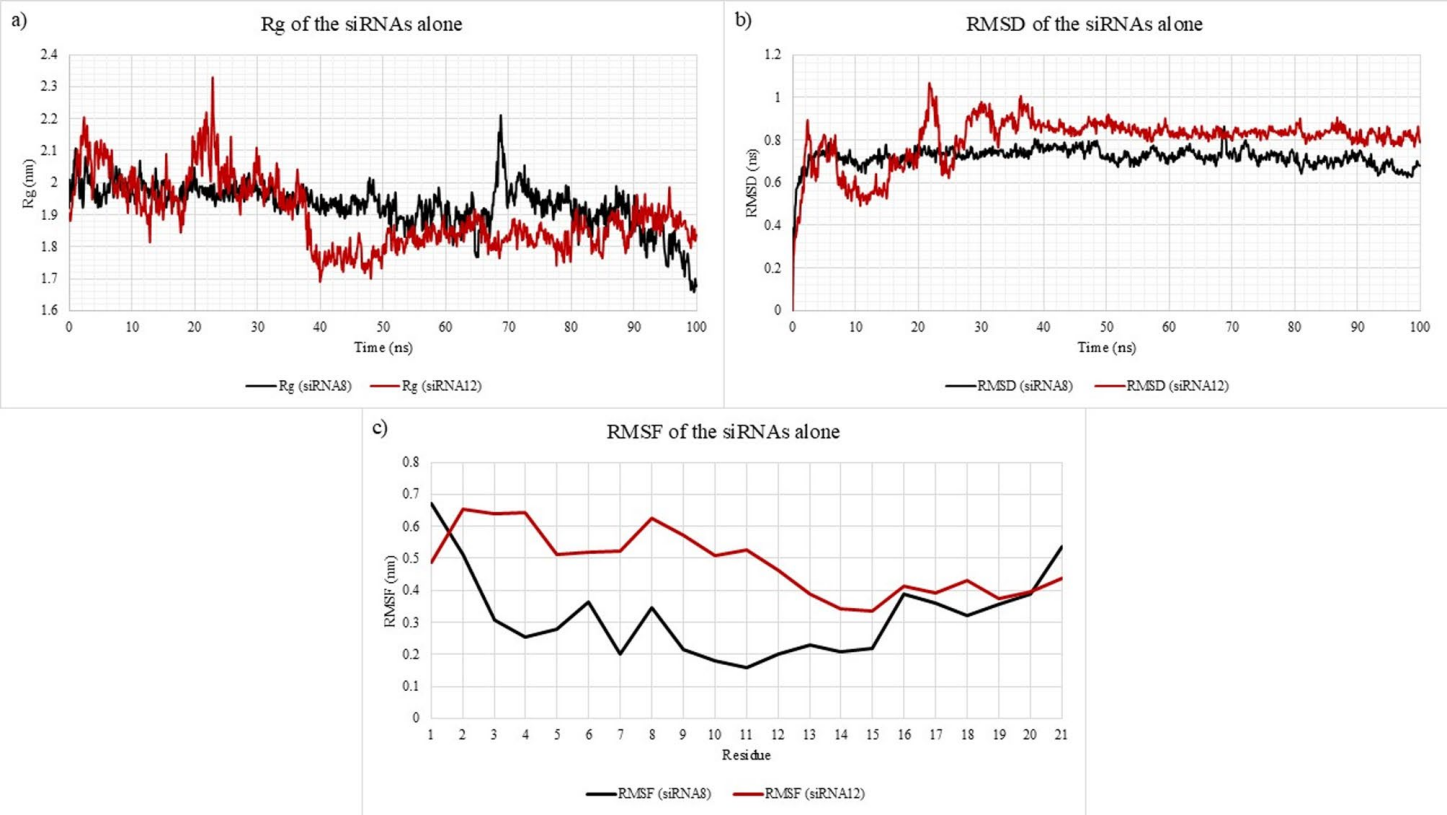
Structural dynamics of siRNAs in isolation, specifically siRNA8 (black) and siRNA12 (red). (**a**) Radius of gyration (Rg) profiles of siRNA8 and siRNA12 over a 100 ns molecular dynamics simulation, indicating overall compactness and structural fluctuations. (**b**) Root mean square deviation (RMSD) of siRNA8 and siRNA12 during the simulation period, reflecting the stability and conformational drift of each siRNA relative to their initial structures. (**c**) Root mean square fluctuation (RMSF) per nucleotide residue for siRNA8 and siRNA12, demonstrating residue-level flexibility differences. siRNA12 exhibits higher structural deviations and flexibility compared to siRNA8, suggesting lower conformational stability.

To further investigate the inherent behavior of the siRNAs independent of AGO2, the siRNA strands were simulated in isolation following complex removal. This allowed for the evaluation of their individual structural stabilities. The Rg analysis (Fig. 7a) showed that siRNA12 had a lower average Rg (~ 1.896 nm) than siRNA8 (~ 1.931 nm), indicating slightly higher compactness and potentially better intrinsic stability. However, a visual assessment of the Rg trajectories suggests that siRNA8 exhibited fewer and more gradual fluctuations over time, implying relatively greater structural consistency. RMSD profiles (Fig. 7b) supported this observation, where siRNA8 exhibited a lower and more stable RMSD (~ 0.72 nm) than siRNA12 (~ 0.8 nm), whose profile showed noticeably greater fluctuations. The RMSF plot (Fig. 7c) demonstrates that siRNA12 exhibited consistently higher residue-level fluctuations than siRNA8 across nearly the entire strand, particularly within the 5′ end (residues 1–8). In contrast, siRNA8 showed notably reduced flexibility, with RMSF values dropping below 0.3 nm from residue 2 onward, indicating a comparatively more stable and rigid conformation in the unbound state. Collectively, these findings indicate that although siRNA12 may be slightly more compact in structure, siRNA8 is overall more conformationally stable, which may be advantageous for maintaining guide strand integrity and consistent behavior during AGO2 loading and target engagement.

The RMSD, radius of gyration (Rg), potential energy (PE), and root mean square fluctuation (RMSF) profiles for both the AGO2-siRNA complexes and the siRNA-only systems plateaued within the first 5–10 ns and remained stable thereafter, indicating equilibrium well before the midpoint of the 100 ns production run. From ~ 40 ns onwards, all monitored parameters fluctuated only within narrow ranges: the AGO2-siRNA12 complex and both siRNA-only simulations exhibited clear convergence, while the AGO2-siRNA8 complex displayed only mild local rearrangements, with stable Rg, PE, and domain-level RMSF values throughout. These stability patterns confirm that the relevant conformational space for the bound state was thoroughly sampled within the available simulation time.

Given this rapid and sustained convergence, additional per-complex replica simulations were deemed unnecessary for the present qualitative, bound-state comparison. Our aim was to compare the relative stability and structural behavior of two pre-formed AGO2–siRNA complexes (siRNA8 and siRNA12) and their corresponding siRNA-only systems, rather than to estimate free energies, kinetic rates, or capture rare, large-scale conformational transitions. Under these conditions, once equilibrium is established and sampling extends far beyond the equilibration period, as is the case here with ~ 90 ns of continuous post-equilibration dynamics, further replicas predominantly re-sample the same equilibrium basin without altering qualitative rankings or mechanistic interpretations. The key trends that underpin our conclusions, namely greater flexibility and lower PE for siRNA8 versus greater rigidity for siRNA12, were reproduced across multiple independent observables and in both bound and unbound contexts, providing internal consistency analogous to the confirmatory role of replicas. This methodological choice is consistent with recent AGO/AGO2 and siRNA/miRNA MD studies in which single, converged ~ 100 ns production trajectories, without per-complex replicas, have been used for bound-state stability assessments and docking-hit evaluation (Lessel et al., 2020^44^; Imanpour et al., 2023^45^; Mallick et al., 2019^46^; Bhandare & Mahfuz et al., 2022^47^). By contrast, multi-replica, multi-microsecond simulations reported in the literature typically address broader, discovery-oriented questions, such as apo-AGO paralog comparisons or investigations of slow domain rearrangements, which differ fundamentally in scope and requirements from the present ranking-focused analysis.

An important consideration in molecular dynamics studies is whether the chosen simulation length matches the timescales of the motions relevant to the research question. Longer simulations are typically required when studying unbound proteins or aiming to capture rare, large-scale conformational transitions. For example, Kakoulidis et al. (2025)^48^ investigated unbound AGO proteins to observe interdomain motions that can occur on the microsecond scale, justifying multi‑microsecond trajectories. In contrast, our study began with pre‑assembled, bound AGO2–siRNA complexes and focused on local rearrangements at the RNA–protein interface, which occur on nanosecond‑to‑tens‑of‑nanoseconds timescales. Once these complexes are equilibrated, additional replicas or substantially longer trajectories would predominantly re sample the same bound-state ensemble without altering the qualitative trends observed. Therefore, the 100 ns simulations used here are consistent with established practice for equilibrated protein–RNA complexes, providing sufficient sampling for robust qualitative comparison while avoiding unnecessary computational cost.

In summary, the molecular dynamics analyses indicate that the AGO2-siRNA8 complex demonstrates superior thermodynamic stability, as reflected by its lower PE profile while maintaining high conformational flexibility, evident from its better trends in the Rg, RMSD and RMSF profiles. These specific features may likely confer greater functional adaptability, which is necessary for dynamic processes such as RISC loading and catalysis of cleavage of target mRNA. On the other hand, the AGO2-siRNA12 complex maintains a more rigid and compact structure, with lower structural deviation and reduced residue-level fluctuations, indicating reduced ability of structural adaptation during its functional process. The isolated siRNA analysis further reinforces the suitability of siRNA8, highlighting its higher standalone stability and lower fluctuation profile as compared to siRNA12. Overall, these findings suggest that siRNA8 may serve as a more functionally compatible candidate for AGO2 loading and potentially enhance gene silencing efficacy.

## Materials and methods

### Dataset collection

The complete coding regions (CDS) of the human mRNA sequence for GPR10 was retrieved from the National Center for Biotechnology Information’s (NCBI) Nucleotide database^22^. This sequence was given as an input for in-silico tools to design and collate the potential siRNA candidates which could silence the GPR10 gene. To perform molecular docking and molecular dynamics, the 3-dimensional structure of the Argonaute 2 protein was obtained from the AlphaFold Protein Structure Database^23,24^.

### Designing candidate siRNAs for GPR10 silencing

To ensure a rational and biologically meaningful selection, each stage of siRNA candidate exclusion was based on standard computational filtering criteria that are well-established in literature for maximizing silencing efficiency and specificity. These included sequence-based design rules (Ui-Tei, Amarzguioui, and Reynolds), GC content thresholds, seed duplex stability, off-target BLAST filtering, mRNA accessibility, thermodynamic favorability, and siRNA-mRNA duplex binding energy. Similar multi-step pipelines have been widely adopted in recent siRNA design studies for disease targets^49,50^. The final shortlist therefore represents not an arbitrary or obvious remainder, but a set of robust candidates that pass stringent performance and specificity filters, offering the highest likelihood of functional silencing in downstream experimental validation.

A list of predicted siRNA candidates corresponding to the human GPR10 mRNA was generated by using the siDirect version 2.0, a web server for designing siRNAs with high target specificity and silencing efficiency^27^. The FASTA format of the human mRNA sequence for GPR10 was entered as an input for this. The predicted siRNAs were set to satisfy all three functional siRNA design algorithms which were: the Ui-Tei^28^Reynolds^29^ and Amarzguioui^30^ algorithms. To minimize off-targeting of the siRNAs, the maximum melting temperature of the seed-duplex was set to 21.5 °C. The GC content of the siRNAs was set to a range of 30–55% to ensure a balance of duplex stability and efficient strand separation during RISC loading. Furthermore, this ensures the resulting siRNAs will have an optimal binding affinity with the target mRNA without sacrificing on its specificity, thereby increasing cleavage efficiency. A high GC content will enable stronger binding affinity with the mRNA but it introduces the risk of unintended binding to non-target mRNAs due to strong hybridization.

### Structural and thermodynamic analysis of siRNA

To predict the secondary structures of the single stranded siRNA candidates, the MaxExpect tool from RNAstructure web server (Version 6.0.1, Mathews Lab, University of Rochester Medical Center)^32^ was employed. It is a tool for predicting RNA or DNA secondary structures which are optimized to include base pairs with the highest probability of being correct. For this study, the 23-nucleotide long siRNA guide strand was given as an input while maintaining the other parameters to default values. MaxExpect then provides the maximum expected accuracy structure, which is defined as the structure with the highest sum of correctly predicted base pairing probabilities. This approach has been shown to more accurately reflect experimentally determined RNA secondary structures than traditional minimum free energy-based methods^51^.

The DuplexFold tool from the RNAstructure web server^32^ was utilized to predict the hybridization of two separate RNA strands and to evaluate the energetic stability of their interaction. This tool computes the most thermodynamically stable structures by evaluating all possible intermolecular base pairs between the two nucleotide strands while disallowing intramolecular base pairing, thereby determining the configuration with the minimum free energy (ΔG). Designed siRNA guide strands were aligned to their complementary target regions within the mRNA sequence using DuplexFold to model RNA-RNA duplex formation. Predictions were performed under default conditions at 37 °C with standard ionic strength (1 M NaCl), and the output included the predicted intermolecular base-pairing structure and the associated minimum free energy of the duplex.

The secondary structure formed by each of the single stranded siRNA candidates was analysed using Mfold tool^33^ from the Unified Nucleic Acid Folding (UNAFold) Web Server^52^. The tool calculates the most thermodynamically stable structures possible for the given nucleotide sequence, mainly through minimization of free energy using dynamic programming algorithms and established thermodynamic data. The nucleotide sequence of each siRNA guide strand was input in linear single-stranded RNA format, as appropriate for siRNA structure prediction. With default parameters such as the fixed folding temperature of 37 °C and 1 M NaCl ionic concentration, the associated free energy of folding upon secondary structure formation was predicted and used to assess the stability of each siRNA.

The DI-Nucleic Acid hybridization and melting prediction (DINAmelt) tool^34^from the UNAFold Web Server^50^was utilized to predict the melting behaviour of the hybridized siRNA and mRNA strand. Nearest-neighbor thermodynamic models, which consider the stability of each base pair with respect to its adjacent pairs, are used for this prediction. It simulates the thermal denaturation process and provides equilibrium melting profiles as a function of temperature along with melting temperature values such as Tm (Conc) and Tm (Cp), which are crucial parameters for evaluating the effectiveness of siRNAs. The single nucleotide sequence of each of the siRNA candidates and their corresponding target mRNA was given as an input. Using the energy minimization model and default parameters the melting temperature values were calculated for each duplex to help evaluate the most efficient siRNAs.

The OligoWalk module available through the RNAstructure web server^36^ was used to predict the silencing efficacy of the designed siRNA guide strands. This tool allows for the evaluation of each siRNA’s ability to bind effectively to its target site within the full-length GPR10 mRNA sequence. Each guide strand was submitted individually, and the analysis was performed using default parameters. OligoWalk predicts siRNA efficacy by considering factors such as duplex stability, target site accessibility, and the overall energy profile of binding. For each siRNA, the tool generates an efficacy score expressed as a percentage, which reflects the likelihood of successful gene silencing. These percentage scores were used to rank the siRNA candidates and identify the most effective guide strands for further analysis.

The GC content of each siRNA guide strand was calculated using the Oligonucleotide Properties Calculator provided by Bio-Synthesis Inc^35^. The nucleotide sequences were entered in single-stranded RNA format, and the GC percentage was computed automatically by the tool to assess duplex stability and ensure optimal base pairing characteristics. The thermodynamic stability of each siRNA guide strand was also estimated using the same tool^35^. The Gibbs free energy (ΔG) values, indicating the spontaneity of siRNA folding, were calculated under standard conditions set to 1 M NaCl, 25 °C, and pH 7. These conditions, which represent the default parameters of the Oligonucleotide Properties Calculator, may differ from those used in the molecular dynamics (MD) simulations. However, the ΔG values (expressed in kcal/mol) were used solely for the relative comparison of thermodynamic stability among siRNA candidates during the selection process and were not intended to replicate physiological environments.

### Docking of siRNA with Argonaute 2 protein

To evaluate the binding interaction between the designed siRNA candidates and the Argonaute 2 (AGO2) protein, molecular docking was performed using the HDOCK web server^37^a hybrid docking algorithm that supports protein-RNA docking. The three-dimensional structure of AGO2 was obtained from the AlphaFold Protein Structure Database^24^while the siRNA guide strands were modelled as linear RNA molecules.

For each docking run, the AGO2 structure was provided as the receptor and the siRNA guide strand as the ligand. The docking was performed in RNA-protein mode using default parameters. HDOCK generates docking models by combining template-based modeling and free docking strategies and ranks them based on a knowledge-based scoring function. The top-ranked docking poses were retrieved based on docking score, which represents the predicted binding affinity and interaction potential between AGO2 and the siRNA candidate.

The resulting docking complexes were visualized using PyMOL^38^ to examine how each siRNA was positioned within the AGO2 binding pocket.

### Molecular dynamics simulation

Molecular dynamics (MD) simulation was performed using GROMACS version 2023.1^39^ to evaluate and compare the stability of the selected siRNA-AGO2 complexes. The input files for the simulations were prepared and generated using CHARMM-GUI’s Solution Builder^53,54^ modules. CHARMM-GUI^55^ is a web server designed to facilitate and standardize the preparation of biomolecular simulation systems using the various force fields. In our study, the CHARMM36 force field was used.

The input file preparation began with the PDB Reader module, which processed the input structure by assigning proper protonation states, resolving any missing atoms, and converting it into CHARMM-compatible format. Subsequently, the Solvator module was used to embed the complex in a cubic box filled with TIP3P water molecules with a 10.0Å buffer distance and ion concentration (0.15 M KCl by default) to mimic physiological conditions. Monte Carlo simulations were used to determine the positions of the ions placed, thereby ensuring electroneutrality. CMAP corrections were automatically applied as part of the CHARMM36 force field parameters during system preparation. Standard protonation states of titratable residues were assigned by CHARMM-GUI based on default pKa predictions at pH 7.0, and no manual modifications were made.

Following solvation, all necessary input files were generated to be compatible with GROMACS, such that energy minimization, equilibration, and production runs can directly be performed. These input files included predefined protocols for steepest descent minimization, equilibration under constant volume (NVT) and constant pressure (NPT) ensembles, and unrestrained production dynamics, corresponding to the energy minimization, equilibration, and production phases, respectively.

After the necessary input files were generated, a 100 ns production run was carried out for each of the siRNA-AGO2 complexes, with the run length consistent with previous MD studies of AGO2-siRNA complexes. This timescale was chosen to allow sufficient sampling of binding interactions and conformational stability. Following this, the analysis was performed where the resulting root-mean-square deviation (RMSD), root-mean-square fluctuation (RMSF), radius of gyration, and potential energy profiles were generated using standard GROMACS utilities and were compared between the siRNA-AGO2 protein complexes.

## Conclusions

This study presents a rational, in silico approach to designing siRNAs targeting GPR10, a key molecular driver implicated in uterine fibroids pathogenesis. Through a comprehensive filtering pipeline incorporating thermodynamic profiling, secondary structure modelling, off-target analysis, molecular docking, and molecular dynamics simulations, we identified 2 siRNAs down from 275 potential candidates, which were siRNA8, and siRNA12 as lead candidates with strong binding affinities to AGO2 and high predicted silencing efficacy. This stepwise exclusion was conducted systematically according to established guidelines to ensure that the final shortlisted siRNAs represent the most robust and specific candidates, rather than arbitrary selections. Among these, siRNA8 emerged as the most promising molecule, exhibiting superior thermodynamic stability and enhanced conformational flexibility in complex with AGO2, attributes that may facilitate more efficient RISC loading and repeated efficient target mRNA cleavage. In contrast, siRNA12 displayed a more rigid and compact structure, suggesting potentially limited adaptability during RNA-induced silencing. Collectively, these findings support siRNA8 as a highly favourable candidate for therapeutic RNAi-based silencing of GPR10 in Uterine Fibroids.

To validate the silencing potential of siRNA8, future work should involve in vitro studies in uterine fibroid cell lines to confirm GPR10 knockdown at both the transcript and protein levels. Furthermore, in vivo validation using fibroid xenograft or appropriate animal models will be essential to assess therapeutic efficacy, specificity, biodistribution, and safety. However, successful clinical translation also requires overcoming major barriers to siRNA delivery, including susceptibility to nuclease degradation, limited membrane permeability, and inefficient endosomal escape^56^. To address these challenges, several delivery strategies have shown promise. Lipid nanoparticles (LNPs) have emerged as a leading non-viral delivery platform due to their ability to encapsulate siRNA, protect it from degradation, and promote cytoplasmic delivery through endosomal disruption mechanisms such as the proton sponge effect and membrane fusion. FDA-approved LNP-based siRNA drugs, including patisiran and givosiran, demonstrate the clinical viability of this approach^43^. Polymer-based nanoparticles and dendrimer systems also offer tunable physicochemical properties for optimized delivery, while conjugation strategies using targeting ligands such as peptides, antibodies, or N-acetylgalactosamine (GalNAc) enable tissue-specific targeting and enhanced uptake in specific cell types^57^.

Therefore, incorporating these delivery innovations with rational siRNA design can significantly improve pharmacokinetics, intracellular trafficking, and gene-silencing potency. Thus, integrating siRNA8 with a targeted delivery platform optimized for uterine tissue could pave the way for a clinically viable, non-hormonal RNAi therapy for uterine fibroids.

## Supplementary Information

Below is the link to the electronic supplementary material.

Supplementary Material 1

## Data Availability

The GPR10 mRNA sequence (accession number: NM\_004248.3) is available on NCBI Nucleotide database. The 3D structure of Argonaute 2 (AGO2) is available on the AlphaFold Protein Structure Database (UniProt Accession Number: Q9UKV8).
