# Supplementary material for: Molecular dynamics simulations based siRNA design against GPR10 reveals stable RNAi therapeutics for hormone-dependent uterine fibroids
Source: Sci Rep. 2025 Aug 28;15:31708. doi: 10.1038/s41598-025-16936-z (PMC12394437; doi:10.1038/s41598-025-16936-z)
Supplement: Supplementary file 1 — Supplementary Material 1 [file 41598_2025_16936_MOESM1_ESM.docx]

| ***siRNA name*** | ***mRNA target position*** | ***mRNA target sequence (5’→3’)*** | ***siRNA guide strand (5’→3’)*** | ***siRNA passenger strand (5’→3’)*** | ***Seed-Duplex Stability*** | | ***GC % content*** | ***Tm***  ***(Conc)*** | ***Tm(Cp)*** | ***Thermodynamic Constant ΔG (kcal/mol)*** | ***Free Energy of Binding (kcal/mol)*** | ***Free Energy of Folding (kcal/mol)*** | ***siRNA Efficacy (%)*** |
| --- | --- | --- | --- | --- | --- | --- | --- | --- | --- | --- | --- | --- | --- |
|  |  |  |  |  | ***Guide Tm (°C)*** | ***Passenger Tm(°C)*** |  |  |  |  |  |  |  |
| siRNA3 | 1514-1536 | GCCCTTGAAGGACAAACAAAACT | UUUUGUUUGUCCUUCAAGGGC | CCUUGAAGGACAAACAAAACU | 5.6 | 16.6 | 43% | 88.6°C | 89.8°C | -26.1 | -35.2 | 2 | 93.2486 |
| siRNA6 | 1861-1883 | CACCTACAAGCTCCCTTTAGAAA | UCUAAAGGGAGCUUGUAGGUG | CCUACAAGCUCCCUUUAGAAA | 15.8 | 16.4 | 48% | 90.9°C | 92.1°C | -25.9 | -38.7 | 1.7 | 91.0699 |
| **siRNA8** | **2031-2053** | **CTGTGTATGTGTGTTTGTGTATG** | **UACACAAACACACAUACACAG** | **GUGUAUGUGUGUUUGUGUAUG** | **19.3** | **20.4** | **38%** | **84.9°C** | **86.1°C** | **-24** | **-33.7** | **1.9** | **93.4435** |
| siRNA10 | 2308-2330 | CCCACATGATGTGTAGATCAAAC | UUGAUCUACACAUCAUGUGGG | CACAUGAUGUGUAGAUCAAAC | 21.4 | 20.5 | 43% | 86.7°C | 87.8°C | -24.7 | -36.8 | 1.8 | 95.2393 |
| **siRNA12** | **3722-3744** | **CCGAATGAAGCTTTAGTTGTACC** | **UACAACUAAAGCUUCAUUCGG** | **GAAUGAAGCUUUAGUUGUACC** | **19** | **12** | **38%** | **82.9°C** | **84.2°C** | **-24.8** | **-32.9** | **1.8** | **96.1845** |
| siRNA13 | 3780-3802 | CGGTGTTACATGCCATACTTAGA | UAAGUAUGGCAUGUAACACCG | GUGUUACAUGCCAUACUUAGA | 11.6 | 20.4 | 43% | 86.8°C | 86.7°C | -25.5 | -35.8 | 1.7 | 93.0229 |
| siRNA14 | 3782-3804 | GTGTTACATGCCATACTTAGAGA | UCUAAGUAUGGCAUGUAACAC | GUUACAUGCCAUACUUAGAGA | 11.3 | 13.5 | 38% | 84.8°C | 86.0°C | -23.6 | -34.6 | 1.7 | 92.2715 |
| siRNA17 | 4245-4267 | GAGCAAAATCTAGTCTCAAAAAA | UUUUGAGACUAGAUUUUGCUC | GCAAAAUCUAGUCUCAAAAAA | 20.4 | 7.4 | 33% | 83.1°C | 84.4°C | -23.2 | -31.8 | 1.6 | 94.9888 |
| siRNA18 | 4973-4995 | AAGGATATTCCAGTTCCTATAAC | UAUAGGAACUGGAAUAUCCUU | GGAUAUUCCAGUUCCUAUAAC | 19.9 | 11.6 | 33% | 85.5°C | 86.6°C | -22.5 | -33.3 | 1.8 | 94.38 |
| siRNA23 | 5273-5295 | GTCTTTGCTATGTGTGTAAAATG | UUUUACACACAUAGCAAAGAC | CUUUGCUAUGUGUGUAAAAUG | 14.7 | 19.7 | 33% | 83.9°C | 85.2°C | -23.3 | -31.4 | 1.8 | 94.8632 |

**Supplementary Table:** Biophysical properties and structural-thermodynamic parameters of the top 10 siRNA candidates out of 275 generated via siDirect targeting GPR10 mRNA.
